# Genetic factors and manganese-induced neurotoxicity

**DOI:** 10.3389/fgene.2014.00265

**Published:** 2014-08-04

**Authors:** Pan Chen, Nancy Parmalee, Michael Aschner

**Affiliations:** Department of Molecular Pharmacology, Albert Einstein College of MedicineBronx, NY, USA

**Keywords:** manganese, manganism, Parkinson’s disease, neurotoxicity

## Abstract

Manganese (Mn), is a trace metal required for normal physiological processes in humans. Mn levels are tightly regulated, as high levels of Mn result in accumulation in the brain and cause a neurological disease known as manganism. Manganism shares many similarities with Parkinson’s disease (PD), both at the physiological level and the cellular level. Exposure to high Mn-containing environments increases the risk of developing manganism. Mn is absorbed primarily through the intestine and then released in the blood. Excessive Mn is secreted in the bile and excreted in feces. Mn enters and exits cells through a number of non-specific importers localized on the cell membrane. Mutations in one of the Mn exporters, SLC30A10 (solute carrier family 30, member 10), result in Mn induced toxicity with liver impairments and neurological dysfunction. Four PD genes have been identified in connection to regulation of Mn toxicity, shedding new light on potential links between manganism and PD.

## INTRODUCTION

Manganese (Mn) is present in biological organisms in trace amounts and is necessary for optimal functioning of multiple enzymes, such as hydrolases, lyases, glutamine synthetase, and superoxide dismutase (SOD; Friberg et al., 1979; Wedler and Denman, 1984). Mn is also an essential cofactor for arginase (Roholt and Greenberg, 1956), an enzyme required for elimination of ammonia, as well as SOD, the principle mitochondrial antioxidant enzyme (McCord, 1976). In addition to these essential functions, Mn modulates the immune system, cell adhesion, as well as protein and carbohydrate metabolism (Grinnell, 1984; Smialowicz et al., 1985). Mn is required for proper bone and brain development (Frost et al., 1959; Sandstead, 1986), and Mn deficiency, while rare due to abundant Mn in the diet, can result in birth defects and seizures (Aschner, 2000, 2002). Mn levels are tightly regulated; however, excessive exposure to this metal may result in elevated brain accumulation and subsequent neurodegeneration, referred to as manganism.

Toxic exposures to Mn are most commonly seen in miners (Rodier, 1955), welders (Racette et al., 2001), and individuals working in and living around ferroalloy plants (Lucchini et al., 2007). As observed by T1-weighted magnetic resonance imaging (MRI), excessive levels of Mn accumulate preferentially in the basal ganglia (Inoue et al., 1991; Kulisevsky et al., 1992; Pujol et al., 1993; Butterworth et al., 1995; Weissenborn et al., 1995; Lucchini et al., 2000). The symptoms of idiopathic Parkinson’s disease (PD) overlap considerably, but not completely, with manganism. In PD, the earliest symptoms include loss of smell and sleep disorders, followed by difficulty with balance, gait disturbance, and unilateral resting tremor (Parkinson, 2002). Presentation varies considerably among patients. The disease typically progresses to include bradykinesia, rigidity, postural instability, masked facial expression, and difficulty with speech and swallowing. PD patients may develop visual symptoms, including a decline in visual perception, disturbances of visuospatial orientation, facial recognition problems, and chronic visual hallucinations (Armstrong, 2011). A substantial portion of PD patients also experience depression and/or dementia. Neuropathologically, PD is characterized by the loss of dopaminergic (DAergic) neurons in the substantia nigra (SN) pars compacta, and the presence of Lewy bodies, neuronal protein aggregates that are composed of alpha-synuclein and other proteins (Jellinger, 2012).

## NEUROTOXICITY INDUCED BY Mn EXPOSURE

### HISTORY AND SYMPTOMS OF MANGANISM

Mn toxicity was first described by Couper (1837) reporting his observations of five manganese ore crushers with muscular weakness, paraplegia, tremor, whispering speech, and a tendency to lean forward while walking. In two of the workers, symptoms progressed even after removal from the Mnore crushing operations. The remaining three workers were removed from Mn exposure at the first signs of muscle weakness in the lower extremities. In these three workers, the symptoms abated following removal from Mn exposure. Manganism was also described by Rodier (1955) in a group of Moroccan miners, by Mena et al. (1967) in a group of Chilean miners, and by Huang et al. (1989) in six Taiwanese ferromanganese plant workers. Of ∼4000 miners working in three mines in Morocco, Rodier saw 150 cases of manganism, all in workers who spent considerable time underground, presumably with limited ventilation and a high degree of inhalation exposure. In these workers, onset of symptoms occurred after months to years of work. In contrast, Racette (2013) noted that contemporary exposures are much lower, and onset of symptoms tends to occur after years or decades of exposure. Mena described Mn turnover in patients compared to healthy miners using injected ^54^Mn radioisotope and found significantly faster turnover rates in the healthy miners as compared to miners diagnosed with manganism. Huang described six patients who worked in a factory with inadequate ventilation. Follow up studies on these patients indicate that after removal from Mn exposure, their symptoms continued to worsen for approximately ten years, after which their condition remained constant with severe extrapyramidal dysfunction (Huang et al., 1993, 1998, 2007). Early features of manganism include weakness, anorexia, behavioral, and psychiatric disturbances, difficulty sleeping, impotence, and attention disorders. This early stage is characterized by emotional lability that has been termed “manganese madness.” Patients have described an awareness that their behavior is unusual or abnormal, but an inability to control their outward affect. There is some evidence that if Mn exposure is discontinued at an early stage, these symptoms may revert. However, with acute exposure, progression often occurs even after Mn exposure has ceased. MRI shows clearing of Mn from the globus pallidus (GP) in three to six months (Roth, 2009). In some patients with severe exposure, neurodegenerative signs progress even after removal from exposure. Later symptoms include extrapyramidal deficits including bradykinesia, rigidity, difficulty with gait, specifically a dystonic movement of the foot known as “cock walk,” and a tendency to fall backward. Masked facial expressions and micrographia are also present in manganism, as in PD (Couper, 1837; Rodier, 1955; Mena et al., 1967). Tremor in manganism tends to be slight or absent as compared to PD. Manganism patients do not typically respond therapeutically to L-DOPA (Shinotoh et al., 1997; Koller et al., 2004).

### MANGANISM AND PARKINSON’S DISEASE: SIMILARITY AND DIFFERENCES

There is evidence that genes involved in susceptibility to PD are also involved in Mn uptake and homeostasis (discussed in more detail below), leading to the hypothesis that there are common mechanisms of neurodegeneration to manganism and PD. In each cohort, only a subset of exposed individuals developed signs of manganism. It is unknown why some individuals are apparently able to maintain Mn homeostasis in the presence of high exposures, while others accumulate toxic levels of the metal leading to neurodegeneration, or why some individuals improve following cessation of exposure, while others progress. The distinction between manganism and PD is typically made based on exposure history, however, in areas with high levels of naturally or industrially occurring Mn, PD prevalence is higher than in surrounding regions or national registries (Lucchini et al., 2007), indicating that the distinction between PD and manganism may not be entirely clear, especially for chronic, long-term exposures. Understanding the molecular genetics of Mn transport and toxicity may lead to a new understanding of the mechanisms underlying PD.

## FACTORS REGULATING Mn TRANSPORT AND TOXICITY

### ENVIRONMENTAL FACTORS

As the 5th most abundant metal and the 12th most abundant element on the earth, Mn is present in air, soil, and water. Mn is taken up primarily through the intestine and then circulated in the blood (Davis et al., 1993; Finley et al., 1994). Excessive Mn is secreted via the bile, returning to the intestine and excreted in feces (Davis et al., 1993; Malecki et al., 1996). Mn may also enter the human body by inhalation of fumes containing the metal (Jorge da Silva et al., 2008). It is also secreted through urine and sweat, but in smaller amounts (vs. bile; Cohn and Emmett, 1978; Davis et al., 1993).

Given the abundance of Mn in the environment, humans are exposed to different levels of Mn. Rice, nuts, whole grains, and legumes, as well as green vegetables, tea, and certain fruits contain high Mn levels (ATSDR, 2012). Food-borne Mn levels generally supply the basic dietary requirement for humans without causing disease. However, excessive exposure to environmental Mn results in toxicity. Workers in mining, welding, and smelting industries may be potentially exposed to high Mn levels (Criswell et al., 2012; Racette et al., 2012), as well as individuals residing in the vicinity of Mn associated industries. Exposures to high Mn levels have been associated with greater risk for PD (Racette et al., 2012; Smith et al., 2012). Additional exposures to Mn have been documented from gasoline, steel, fireworks, batteries, leather, glass, ceramics, cosmetics, and textiles, as well as certain pesticides and fungicides (such as Maneb and Mancozeb; ATSDR, 2012). In addition to exposure in high Mn containing environments, patients with dysfunctional biliary system (e.g., those requiring parenteral nutrition) are also more susceptible to Mn induced toxicity, as optimal liver function is required for Mn secretion. In addition, infants and children that receive Mn-containing supplements may be at risk to Mn toxicity, as younger individuals tend to absorb and maintain higher Mn levels compared to adults reflecting a greater requirement for Mn in cellular activities (Keen et al., 1986; Zlotkin et al., 1995). Iron (Fe)-deficient individuals are also at a greater risk for Mn poisoning, as the two metals compete for shared transporters (see below). In recent years, Methcathinone abuse has been linked to manganism, due to the presence of Mn as an impurity (derived from potassium permanganate; Sikk et al., 2013).

### GENETIC FACTORS

#### Transporters importing extracellular Mn ( Table 1)

Mn enters cells by various transporters (importers) located at the plasma membrane. These transporters are tightly regulated, maintaining optimal Mn levels in cells. The divalent metal transporter 1 (DMT1) is the primary divalent Mn (Mn^2+^) transporter. It is localized at the plasma membrane (Burdo et al., 2001a) and functions to import Mn^2+^ from the extracellular matrix into cells (Bell et al., 1989; Aschner and Gannon, 1994; Erikson and Aschner, 2002; Garrick et al., 2003; Au et al., 2009). DMT1 is highly expressed in basal ganglia, such as SN, GP, hypothalamic nucleus, and striatum (Williams et al., 2000; Burdo et al., 2001b; Huang et al., 2004), areas that are known to accumulate high Mn levels (Suzuki et al., 1975; Newland et al., 1989; Rose et al., 1999; Ikeda et al., 2000; Jorge da Silva et al., 2008). In addition to DMT1, other transmembrane proteins also import Mn^2+^, including the citrate transporter (Crossgrove et al., 2003), zinc transporters ZIP8 (He et al., 2006; Himeno et al., 2009; Fujishiro et al., 2011), calcium channels (Mason, 1993; Lucaciu et al., 1997; Finley, 1998), the choline transporter (Lockman et al., 2001), dopamine transporter (DAT; Ingersoll et al., 1999), and a cation-transporting ATPase 13A2 (ATP13A2 or Park9; Gitler et al., 2009; Tan et al., 2011). Trivalent Mn (Mn^3+^) enters cells primarily via the transferrin receptor (TfR) mechanism (Morris et al., 1992). Notably, none of these proteins is a specific Mn transporter, and they transport additional metals, such as iron, calcium, zinc, etc. Currently, few human diseases are known to be associated with mutations in these importers, with the exception of Park9 and DMT1. Park9 represents a well-known risk factor for PD. High levels of Fe were found in brain due to altered DMT1 expression (Urrutia et al., 2013). Mutations in DMT1 that impair Fe transport protect rodents against parkinsonism-inducing neurotoxins, such as MPTP and 6-hydroxydopamine (Salazar et al., 2008), consistent with a role for DMT1 in Fe-mediated neurodegeneration in PD. He et al. (2011) reported that the CC haplotype in the DMT1 gene is a possible risk factor for PD in the Han Chinese population. Whether mutations in DMT1 alter Mn levels in the brains of these individuals has yet to be determined.

**Table 1:** Different types of Mn transporters in a cell.

Types of Mn transporters	Name	Localization	Roles in Mn transportation
**Importers**	DMT1, ZIP8, citrate transporter, calcium channels, choline transporter, DAT, ATP13A2, TfR	Plasma membrane	Import extracellular Mn into cytoplasm
**Exporters**	Fpn, SLC30A10	Plasma membrane	Export cytosolic Mn to extracellular matrix
**Intracellular transporters**	Ca^2+^uniporter	Mitochondrial membrane	Import cytosolic Mn into the mitochondria lumen
	Na^+^-independent mechanisms	Mitochondrial membrane	Export mitochondrial Mn to cytosol
	SPCA1	Golgi membrane	Import cytosolic Mninto the Golgi lumen

#### Transporters exporting intracellular Mn ( Table 1)

Cells export excessive Mn from the cytoplasm to the extracellular matrix, as high Mn levels result in cellular toxicity. Recently, ferroportin (Fpn) was recognized as putative Mn exporter. It transports extracellularly both Fe^2+^ and Mn^2+^ (Yin et al., 2010; Madejczyk and Ballatori, 2012). To date, no human diseases have been ascribed to mutations or dysfunction of this protein, indicating that Fpn is unlikely to be the primary Mn exporter. Recently, a gene named SLC30A10 (solute carrier family 30 member 10) was identified in patients with hepatic cirrhosis, dystonia, polycythemia, and hypermanganesemia, concomitant with high Mn brain levels (Quadri et al., 2012; Stamelou et al., 2012; Tuschl et al., 2012). SLC30A10 is localized at the cell membrane, and mutations in this gene either result in early truncation of this protein or amino acid substitution (Quadri et al., 2012; Stamelou et al., 2012; Tuschl et al., 2012). Interestingly, none of the patients were exposed to excessive Mn, yet they displayed symptoms consistent with PD (Quadri et al., 2012; Stamelou et al., 2012; Tuschl et al., 2012). SLC30A10 is the only protein known to cause Mn toxicity when mutated, indicating it may be a primary and a key regulator of Mn export. Our recent data have shown that the wildtype (WT) SLC30A10protein when expressed in in *Caenorhabditis elegans* (*C. elegans*), protects against Mn^2+^ induced lethality and DAergic neurodegeneration, as well as locomotion deficits (basal slowing response, which is regulated by DAergic neurons; unpublished data). It remains unclear whether SCL30A10 is a specific Mn exporter or if it also transports additional metals. Notably, it was originally posited to be a specific zinc transporter (Seve et al., 2004; Bosomworth et al., 2012). Further research is needed to better understand its function and a compound screen could be profitably directed at identifying specific modulators of this transporter, potentially leading to novel therapies in patients with manganism.

#### Intracellular Mn transporters ( Table 1)

Once inside the cell, Mn is transported into various organelles to fulfill its function, thus limiting its potential toxicity from accumulating at high levels in the cytosol. The mitochondria contain the highest Mn levels (Gavin et al., 1999). Mitochondrial SOD requires Mn as a cofactor for optimal function (Borgstahl et al., 1992). The calcium (Ca^2+^) uniporter imports cytosolic Mn^2+^ into the mitochondria lumen (Drahota et al., 1969; Gunter and Puskin, 1972; Gunter et al., 1975), and excessive Mn^2+^ is exported out of mitochondria through sodium (Na^+^)-independent mechanisms (Gavin et al., 1990). The Golgi apparatus can store excessive Mn^2+^ and this is mediated by Ca^2+^/Mn^2+^ ATPase isoform 1 (SPCA1). SPCA1 is localized at the Golgi membrane and imports Mn^2+^ from the cytosol to the Golgi lumen (Murin et al., 2006). Mn in the Golgi is secreted extracellularly through a secretory pathway. Although the nucleus also contains high levels of Mn, the transporters localized to the nuclear membrane remain unknown and future studies are needed to identify these Mn transporters.

#### PD-associated proteins involved in Mn-induced toxicity ( Table 2)

As described above, manganism shares many similar symptoms with PD. ATP13A2 and DAT both are able to transport Mn. Notably mutations in ATP13A2 cause a parkinsonian-like syndrome, Kufor-Rakeb syndrome (KRS). KRS is an autosomal recessive disorder characterized by subacute, juvenile-onset, and is levodopa-responsive. It is unknown whether mutations in ATP13A2 change its affinity for different cations, specifically Mn, resulting in increased Mn accumulation in the brain. DAT removes synaptic dopamine into the presynaptic membrane for recycling. In PD patients, DAT activity is significantly decreased (Sossi et al., 2007). In *C. elegans*, *dat* knockout mutants are resistant to DAergic neurodegeneration induced by Mn exposure, but show a lower survival rate (Benedetto et al., 2010). Mutations in α*-synuclein*, *parkin*, and *DJ-1*, all of which are associated with early onset PD, have also been shown to alter Mn transport (Bornhorst et al., 2014). α*-synuclein* encodes a presynaptic protein involved in synaptic vesicle trafficking and recycling (Gitler et al., 2008; Ben Gedalya et al., 2009). Point mutations (A30P and A53T) or duplication of α*-synuclein* result in loss of DAergic neurons concomitant with oxidative stress, protein aggregation, and Lewy bodies formation (Narhi et al., 1999; Hsu et al., 2000; Masliah et al., 2000; Kuwahara et al., 2006), the hallmarks of PD. *Parkin* encodes an E3 ubiquitin ligase (Shimura et al., 2000) responsible for degradation of abnormal proteins. Mutations in parkin result in DAergic neurodegeneration with increased oxidative stress, yet in the absence of Lewy bodies (Yang et al., 2006). It is known that overexpression of *parkin* is able to decrease α-synuclein protein aggregation (Petrucelli et al., 2002; Yang et al., 2003). *DJ-1* encodes a protein of the peptidase C56 family. It functions as a peroxidase (Andres-Mateos et al., 2007), a chaperone (Shendelman et al., 2004), a metal binding protein (Björkblom et al., 2013), and a regulatory subunit of an RNA binding complex (Hod et al., 1999), thus protecting cells from oxidative stress (Mitsumoto and Nakagawa, 2001; Mitsumoto et al., 2001), α-synuclein aggregation (Zhou et al., 2009), and metal induced cell death (Björkblom et al., 2013). It also regulates androgen receptor-dependent transcription (Takahashi et al., 2001). Mutations in DJ-1 result in increased oxidative stress and DAergic neurodegeneration. Recently, Chakraborty and colleagues found that *pdr-1* (worm homolog of *parkin*) and *djr-1.1* (worm homolog of *DJ-1*) mutants show enhanced Mn accumulation and increased oxidative stress in *C. elegans* and these effects may be reduced by expression of WT human α-synuclein (Bornhorst et al., 2014). Interestingly, WT α-synuclein also protected against DAergic neurodegeneration induced by Mn exposure in *pdr-1* (the homolog of mammalian *parkin/PARK2*) mutant worms (Bornhorst et al., 2014).

**Table 2:** PD-associated proteins involved in Mn-induced toxicity.

PD-associated proteins	Cellular activity	Roles in Mn toxicity
ATP13A2	Inorganic cation transporter	Importing extracellular Mn into cytoplasm
DAT	DA transporter	Importing extracellular Mn into cytoplasm
Parkin	E3 ubiquitin ligase	Enhancing Mn accumulation when mutated
DJ-1	Antioxidant peptidase	Enhancing Mn accumulation when mutated
α-synuclein	Presynaptic protein in synaptic vesicle trafficking and recycling	Reducing Mn accumulation in *parkin* and *DJ-1* mutants
UCH-L1	Ubiquitin ligase	Unknown
NURR1	Transcription factor	Unknown
PINK1	Serine/threonine kinase	Unknown
LRRK2	Protein kinase	Unknown

In addition to the above proteins, there are other PD-associated proteins whose effect on Mn-induced toxicity remains unknown. These PD genes include ubiquitin carboxy-terminal hydrolase L1 (UCH-L1), NUR-Related factor 1 (NURR1), PTEN-INduced Kinase 1 (PINK1) and Leucine-Rich Repeat Kinase 2 (LRRK2). UCH-L1 (or PARK5) is an enzyme expressed in neurons throughout the brain that hydrolyzes C-terminal ubiquitinyl esters to recycle ubiquitin after degradation of misfolded proteins (Wilkinson, 2000; Meray and Lansbury, 2007). Similar to parkin, it also functions as an ubiquitin ligase to prevent protein aggregation (Liu et al., 2002; Gong et al., 2006; Lansbury, 2006). NURR1 is a transcription factor predominantly expressed in central DAergic neurons (Bäckman et al., 1999; Jankovic et al., 2005). It is critical for development and maintenance of the DAergic system as it regulates the expression of tyrosine hydroxylase (TH; Sakurada et al., 1999), DAT (Sacchetti et al., 2001), vesicular monoamine transporter 2 (VMAT2), and aromatic L-amino acid decarboxylase (AADC; Hermanson et al., 2003). PINK1 (or PARK6) is a mitochondrial targeted serine/threonine kinase, with substrates in the cytosol (Zhou et al., 2008). Recent studies indicate that PINK1, together with parkin, is involved in a mitochondrial quality control system to regulate mitochondrial dynamics and function, and protects against cellular stress (Valente et al., 2004; Clark et al., 2006). LRRK2 or PARK8 is a protein kinase expressed preferentially in DAergic neurons (Han et al., 2008). It interacts with parkin in the cytosol and overexpression of either WT LRRK2 or gain-of-function mutants results in DAergic neurodegeneration (Smith et al., 2005). Together, these proteins either interact with parkin or have similar function to parkin. Given that parkin is involved in regulation of Mn toxicity, these PD-related proteins may play a role in Mn-induced toxicity. Future studies could be profitably directed at the studies on how mutations in these genes enhance basal ganglia Mn accumulation.

## CONCLUSION

Mn is required for multiple physiological processes. It is a cofactor for various enzymes and plays important roles in mediating oxidative stress responses, regulation of immune responses, carbohydrate metabolism, energy production, cell adhesion and protein modification (Benedetto et al., 2009). Although Mn deficiency results in birth defects and poor bone development, given its abundant and ubiquitous presence in the environment, Mn overexposure represents a more critical contemporary concern. Excessive accumulation of Mn in the brain may cause DAergic neurodegeneration and manganism, the latter sharing a clinical picture analogous to PD. At the cellular level, excessive Mn levels have been associated with increased oxidative stress, impaired ATP production, protein aggregation and mitochondrial dysfunction. α-synuclein aggregation or Lewy bodies are absent in the brains of manganism patients. Given the similarities between manganism and PD, Mn is considered an important environmental factor in causing sporadic PD, especially given recent discoveries that certain PD genes (*ATP13A2*, *parkin*, *DJ-1*, and α*-synuclein*) are involved in Mn transport and/or toxicity.

Genetic factors associated with Mn toxicity remain to be explored. Several Mn importers have been identified; however, none have mutations associated with Mn related diseases. Compared to the plethora of Mn importers, knowledge about Mn exporters is limited. Fpn and SCL30A10 are the only two exporters identified to date. Among all known Mn transporters, SLC30A10 appears to be a key regulator of Mn homeostasis, given that mutations in this genecan result in manganism in the absence of overexposure to this metal. SLC30A10 localizes at the cell membrane, exports Mn from cells and affords protection in various experimental models.

Several PD associated genes, namely, either transport Mn (*ATP13A2*; Gitler et al., 2009; Tan et al., 2011) or mediate Mn-induced toxicity (*parkin*, *DJ-1*, and α*-synuclein*; Bornhorst et al., 2014). These results tightly correlate PD with Mn toxicity, supporting the idea of Mn as an environmental factor of PD. It will be of particular interest to study brain Mn levels in PD patients carrying mutations in these genes. Given the similarity between manganism and PD, it is possible that other well known PD genes (UCH-L1, NURR1, PINK1, and LRRK2) also play a role in regulating Mn homeostasis. Future studies should be focused on the relationship between PD-associated proteins and their potential to alter Mn transport and/or toxicity.

## Conflict of Interest Statement

The authors declare that the research was conducted in the absence of any commercial or financial relationships that could be construed as a potential conflict of interest.
